# Time-resolved two-dimensional profiles of electron density and temperature of laser-produced tin plasmas for extreme-ultraviolet lithography light sources

**DOI:** 10.1038/s41598-017-11685-0

**Published:** 2017-10-02

**Authors:** Kentaro Tomita, Yuta Sato, Syouichi Tsukiyama, Toshiaki Eguchi, Kiichiro Uchino, Kouichiro Kouge, Hiroaki Tomuro, Tatsuya Yanagida, Yasunori Wada, Masahito Kunishima, Georg Soumagne, Takeshi Kodama, Hakaru Mizoguchi, Atsushi Sunahara, Katsunobu Nishihara

**Affiliations:** 1 Interdisciplinary Graduate School of Engineering Sciences, Kyushu University, 6-1 Kasugakoen, Kasuga, 816-8580 Fukuoka, Japan; 2 Gigaphoton Inc., 400 Yokokurashinden, Oyama, 323-8558 Tochigi, Japan; 3 Center for material under extreme environment (CMUXE), School of Nuclear Engineering, Purdue University, 500 Central Drive, West Lafayette, 47907 IN U.S.A.; 4 Institute of Laser Engineering, Osaka University, 2-6 Yamadaoka, Suita, 565-0871 Osaka, Japan

## Abstract

Time-resolved two-dimensional (2D) profiles of electron density (*n*_e_) and electron temperature (*T*_e_) of extreme ultraviolet (EUV) lithography light source plasmas were obtained from the ion components of collective Thomson scattering (CTS) spectra. The highest EUV conversion efficiency (CE) of 4% from double pulse lasers irradiating a Sn droplet was obtained by changing their delay time. The 2D-CTS results clarified that for the highest CE condition, a hollow-like density profile was formed, i.e., the high density region existed not on the central axis but in a part with a certain radius. The 2D profile of the in-band EUV emissivity (η_EUV_) was theoretically calculated using the CTS results and atomic model (Hullac code), which reproduced a directly measured EUV image reasonably well. The CTS results strongly indicated the necessity of optimizing 2D plasma profiles to improve the CE in the future.

## Introduction

Extreme-ultraviolet lithography is a promising technology for high-volume manufacturing of next-generation semiconductor devices^1–3^. A carbon dioxide (CO_2_) drive laser and a tin droplet target are used as an efficient extreme-ultraviolet (EUV) light source^4,5^. One of the primary challenges involves the improvement of the conversion efficiency (CE) from laser energy to in-band EUV energy at a wavelength of λ = 13.5 nm (2% full-bandwidth)^5,6^. Debris reduction is also a crucial problem for commercial usage^7^. Therefore, mass-limited targets, such as small tin droplets with a diameter on the order of 20 μm, have been introduced^8^. However, it is desirable to have a large EUV plasma volume within the etendue limits to achieve a large EUV emission^9^. A double-pulse irradiation scheme is proposed, where a pre-pulse laser expands a small tin droplet, and the main laser irradiates when it reaches a size of approximately 300 μm in diameter^7,8^. A CE of approximately 4% was confirmed using this approach^5^.

A strong correlation has recently been shown between the laser absorption and the CE by changing the delay time between the pre- and main-pulse lasers^10^. However, the reported CE was approximately 2% at the highest. The EUV emissivity strongly depends on plasma parameters, such as electron density (*n*_e_), electron temperature (*T*_e_), and average ion charge state (*Z*)^11–13^. Hence, we developed a collective Thomson scattering (CTS) system to measure these parameters^14–16^. In our previous study, it was confirmed that adequate *n*_e_ and *T*_e_ were achieved in the EUV light source plasmas, but this did not provide optimum plasma conditions for high CE^17^.

This paper determined for the first time that it is important to generate a plasma with an optimal two-dimensional (2D) structure to achieve a large EUV conversion efficiency by adding theoretical analysis to the 2D-CTS data, unlike the contents of our previous paper^17^. That is, it is very important to increase the plasma volume, satisfying the optimum conditions for EUV light emission within the range of the permitted etendue condition.

We specifically showed in this manuscript that the plasma expands to a large radius region, and a hollow-like structure of the optimum plasma efficiently emitting EUV is formed under ideal conditions.

## Results

**Figure 1:**
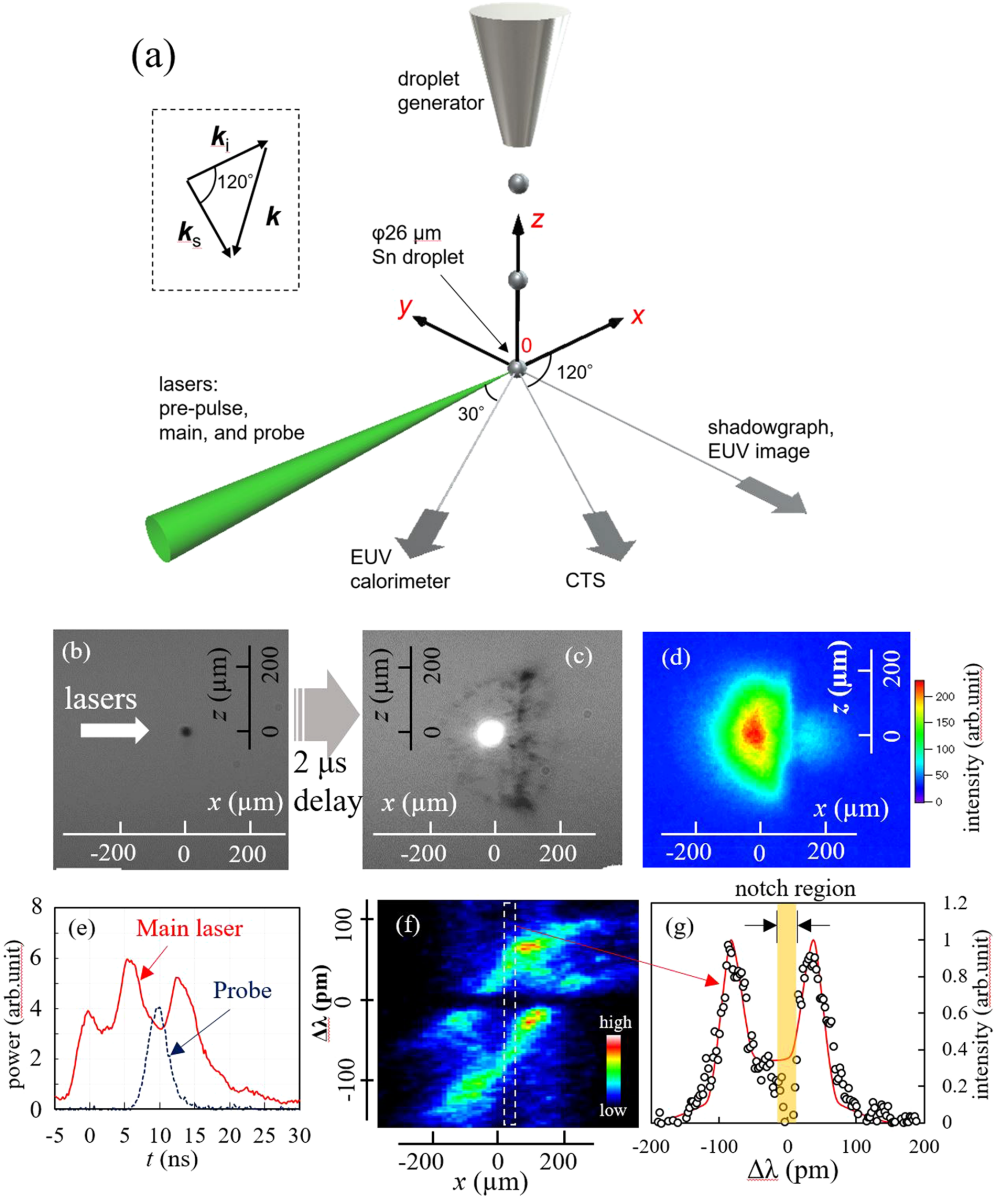
(**a**) Schematic view of the experimental layout. (**b**) Shadowgraph of the initial Sn droplet target (φ = 26 μm) and (**c**) 2 μs after the pre-pulse laser. (**d**) Image of the EUV emission of the 2.0 μs plasma. (**e**) Waveforms of the CO_2_ laser and the CTS probe laser. (**f**) Ion component spectrum measured at time *t* = 10 ns as a function of *x*. (**g**) Ion component spectrum extracted 15 < *x* < 45 μm of (**f**) and its theoretical fitting curve.

Figure 1(a) schematically shows the experimental set-up^17^. A droplet generator supplied the Sn droplet target (diameter: 26 μm) inside a vacuum chamber (<10^−4^ Pa). The pre-pulse and main lasers propagated in the *x* direction and irradiated the droplet. The *x*, *y*, and *z* axes are defined in Fig. 1(a), where the droplets fell in the *z* direction. The origin of the coordinate was set to the initial droplet position before the pre-pulse irradiation. The pre-pulse laser was a Nd:YVO_4_ laser with a 14 ps pulse width [full width at half maximum (FWHM)], a 2 mJ laser energy at 1064 nm, and a spot diameter of 66 µm (1/e^2^ intensity) at the droplet position. The diameter of the 1/e^2^ intensity was used for the laser spot size. The main laser was a CO_2_ laser with a 15 ns pulse width (FWHM), 100 mJ at 10.6 µm, and a spot diameter of 400 µm. Figure 1(b) and (c) show shadowgraphs of the initial tin droplet and that of the expanded tin droplet at 2.0 μs after the pre-pulse irradiation, respectively. Time-integrated, in-band EUV images were observed in the negative *y* axis direction [Fig. 1(d)]. The in-band EUV radiation was measured with an EUV energy sensor located at an angle of 150° from the *x* axis [Fig. 1(a)].

The probe laser for the CTS was the second harmonic of a Nd:YAG laser with a spectral linewidth below <0.1 pm (FWHM) (pulse width: 6 ns FWHM, 3–10 mJ, wavelength λ_0_ = 532 nm, spot diameter of 50 µm in the scattering volume). All three lasers had identical beam paths. The CTS signals were collected by lenses at an angle of 120° from the incident laser direction, focusing on the entrance slit of the spectrometer (20 μm width and 5 mm height) and detected by an intensified charge-coupled device (ICCD) camera (Princeton Instruments, PI-MAX4, 45% quantum efficiency at λ_0_). The *x* axis dimension of the scattering volume was imaged in the slit height direction. Therefore, one-dimensional, spatially resolved measurements were achieved at the same time^14,15^. The CTS measurements were repeated over 30 times at identical experimental conditions. The sufficient reproducibility of the spectra was also confirmed. Regarding the plasma heating by the probe laser, the relative temperature increase (Δ*T*_e_/*T*_e_) was discussed in a previous paper^18^ based on a model considering the absorption by the inverse bremsstrahlung and of the heat transport to the volume surrounding the laser beam during the laser pulse^19,20^. As a result, Δ*T*_e_/*T*_e_ was estimated to be less than 3% for the cases reported here.

The CE for a solid angle of 2π sr was calculated assuming an isotropic distribution of the EUV radiation. Figure 1(e) shows the typical waveforms of the main and probe lasers. As shown in this figure, the time zero (*t = *0 ns) for the CTS measurements was of the first peak of the main laser^17^.

The absolute CE measurements showed that the plasma produced at the delay time of *t*_d_ = 2.0 μs (hereafter referred to as 2.0 μs plasma) had the maximum CE herein (i.e., 4.0%). The CE values for the 1.3 μs and 2.5 μs plasmas decreased to 3.1% and 2.8%, respectively. These three different plasmas were diagnosed by CTS to investigate the relation between the CE and the plasma parameters.

The CTS measurements were performed at 0, 50, 100, 200, and 300 μm in the *y* axis direction [Fig. 1(a)] and at times of *t* = 5, 10, and 15 ns [Fig. 1(e)]. Sufficient symmetry of the plasma along the *y*-axis was confirmed in the complementary CTS measurements in the negative *y*-axis (*y* = −100, −200, and −300 μm) at *t* = 10 ns. The spatial resolutions of the measurements were 13, 50, and 20 μm for the *x*, *y*, and *z* axis directions, respectively [Fig. 1(a)]. These resolutions were determined by the pixel size of the ICCD camera (13 μm), laser spot size (50 μm), and entrance slit width (20 μm). The time resolution was determined by the ICCD camera gate width of 5 ns. Figure 1(f) presents a typical, single-probe laser pulse CTS image for the 2.0 μs plasma at *t* = 10 ns and at *y* = 0 μm. The horizontal axis in the figure is the *x* axis (i.e., laser propagation axis), while the vertical axis is the wavelength difference Δλ from λ_0_. The dark area at Δλ = 0 (within the width of ±14 pm from λ_0_) is the discussed wavelength suppression to reduce the stray light^16^. Figure 1(g) illustrates the spectrum at 15 < *x* < 45 μm of Fig. 1(f) and its fitting curve. We note that the absolute calibration of the CTS system was performed by Rayleigh scattering measurements from nitrogen gas. The vertical (*y*-axis) scan of CTS measurements was performed by moving the focusing position of the probe laser. The absolute calibration of the CTS system was performed at each *y* position. By using the methods described in the following Method section, *n*_e_, *T*_e_ (=*T*_i_), and *Z* were determined as 3.6 × 10^24^ m^−3^, 38 eV, and 12, respectively. The shift of the entire spectrum from λ_0_ was caused by Doppler shift, which reflected a plasma drift velocity in the *k* direction of approximately 1.2 × 10^4^ m/s.

**Figure 2:**
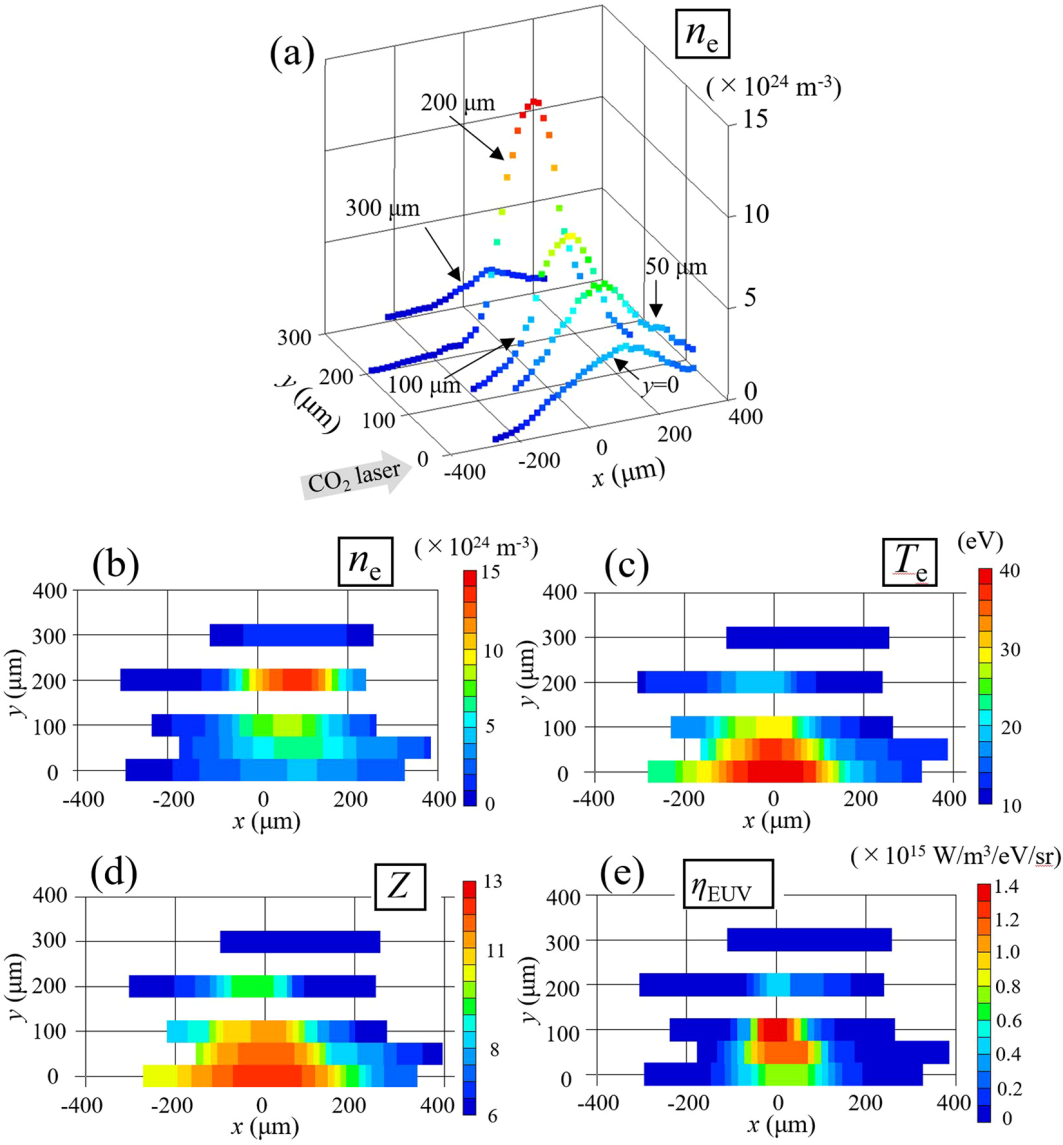
(**a**) Electron density profiles at *y* = 0, 50, 100, 200, and 300 μm at time 10 ns. (**b**,**c** and **d**) Contour plots of electron density, electron temperature, and average ionization, respectively. (**e**) Contour plots of the emissivity obtained from the Hullac code calculation using the CTS data.

Figure 2(a) shows the electron density profiles at different *y* positions from the 2.0 μs plasma at a time of *t* = 10 ns. Figure 2(b)–(d) demonstrate the contour plots of *n*_e_, *T*_e_, and *Z* in the *x–y* planes, respectively. Figure 2(e) presents the local in-band EUV emissivity η_EUV_ (W/m^3^/eV/sr) calculated as a function of *x* and *y* from the measured values of *n*_e_, *T*_e_, and Z and the atomic model based on the Hullac code^12^. Intense η_EUV_ occurred, where a high electron density (≥4 × 10^24^ m^−3^) and a high temperature (≥25 eV) were simultaneously observed. Note that the plasma cut-off density of the CO_2_ laser was 10^25^ m^−3^.

**Figure 3:**
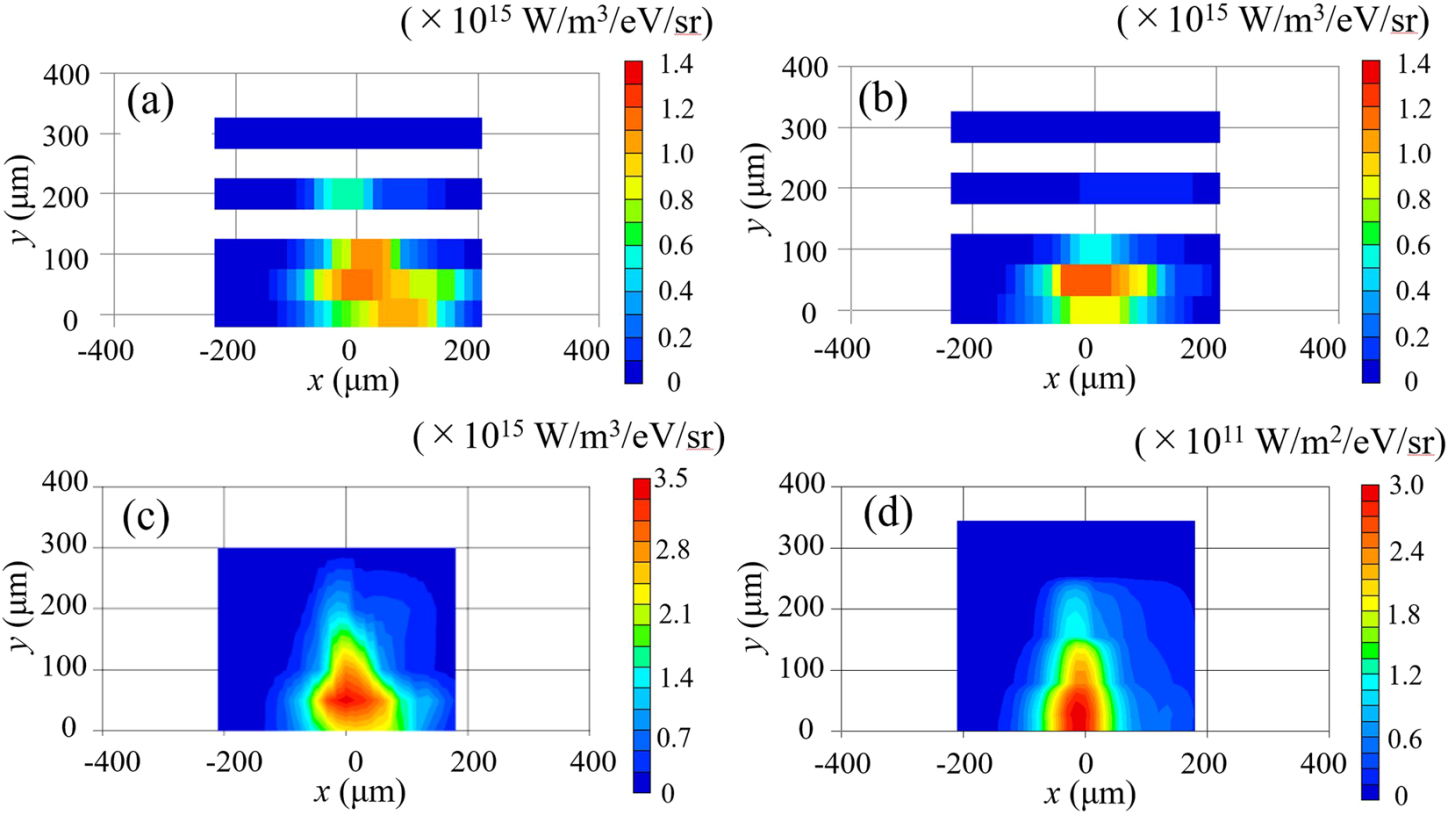
(**a**) Spatial profiles of η_EUV_ at time *t* = 5 ns and (**b**) *t* = 15 ns. (**c**) Sum of η_EUV_ obtained from Figs 2(e) and 3(a) and (b). (**d**) Calculated EUV image obtained by solving the EUV radiation transport with the EUV emissivity shown in Fig. 3(c) with the assumption of the axial symmetry plasma.

Figure 3(a) and (b) show the η_EUV_ profiles of the 2.0 μs plasma measured at time *t* = 5 and 15 ns, respectively. By comparing Fig. 3(a) and (b) with Fig. 2(e), we found that the EUV emission was clearly the strongest at approximately 10 ns. Figure 3(c) shows the sum of the η_EUV_ profiles displayed in Figs 2(e) and 3(a) and (b). The large emissivity of ≥2.5 × 10^15^ W/m^3^/eV/sr occurred at approximately *y*~50–100 μm. The measured EUV image shown in Fig. 1(d) has its maximum at *y* = 0 μm. We calculated the in-band EUV intensity (W/m^2^/eV/sr) represented in Fig. 3(d) from the emissivity profiles [shown in Fig. 3(c)] by solving the radiation transport along the line of sight with taking the plasma self-absorption into account under the assumption of the axial symmetry of the plasma profiles. The plasma self-absorption was calculated from in-band opacity represented in Fig. 15(e) of ref.^12^. The ray trace method [e.g., ref.^21^ and similarly Eq. (8) in ref.^13^] was used to calculate the radiation transport. The profiles shown in Fig. 1(d) (measured) and Fig. 3(d) (calculated using the CTS results) agreed considerably well. For instance, both showed the maximum EUV radiation on the laser axis (*y* = 0). The calculated EUV image [Fig. 3(d)] did not consider the emissivity at times <3 ns. Hence, the EUV image was slightly thinner (narrower) at the laser irradiation side than the measured image in Fig. 1(d).

**Figure 4:**
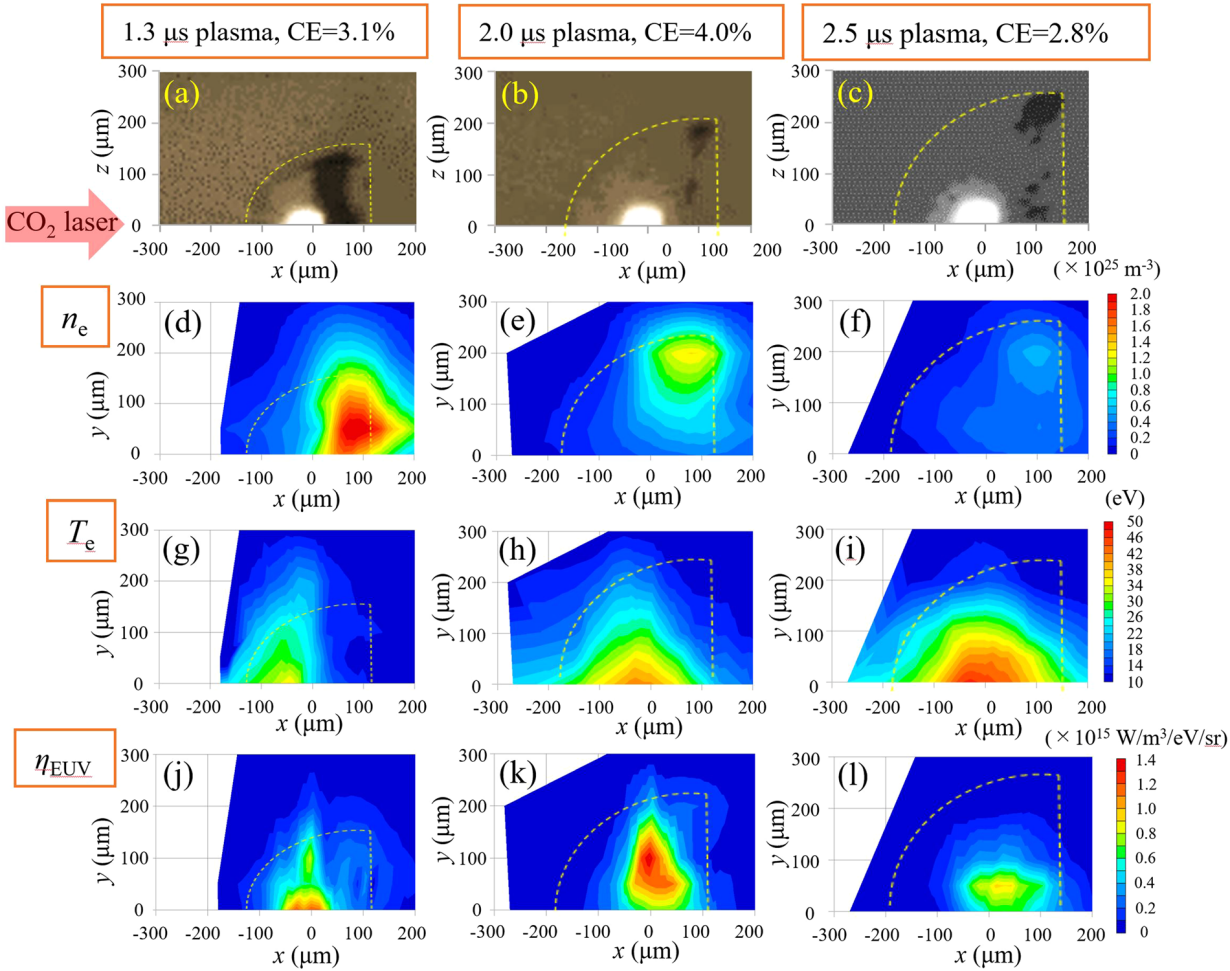
(**a**) Shadowgraph of the 1.3 μs, (**b**) 2.0 μs, and (**c**) 2.5 μs plasmas. Similarly, (**d**)–(**f**), (**g**)–(**i**), and (**j**)–(**l**) show the spatial profiles of *n*_e_, *T*_e_, and η_EUV_ of the 1.3 μs, 2.0 μs, and 2.5 μs plasmas at time *t* = 10 ns, respectively.

Figure 4 represents the shadowgraphs and the spatial profiles of *n*_e_, *T*_e_, and *η*_EUV_ of the three plasma conditions (i.e., *t*_d_ = 1.3, 2.0, and 2.5 μs) measured at *t* = 10 ns. Figure 4(a)–(c) show the shadowgraphs, while Fig. 4(d)–(f) and (g)–(i) demonstrate the *n*_e_ and *T*_e_ profiles, respectively. Figure 4(j)–(l) show the *η*_EUV_ profiles calculated from the corresponding plasma parameters of the three different plasmas. The measured data were interpolated in Fig. 4(d)–(l). Figure 4(e),(h), and (k) use the same data set as in Fig. 2(b),(c), and (e), respectively.

## Discussion

We now discuss the relation between the CE and the spatial profiles of *n*_e_, *T*_e_, and η_EUV_ for the three plasmas shown in Fig. 4(d)–(l). For the case of the 1.3 μs plasma, the high *T*_e_ (>25 eV) region, in which the tin ion can emit the intense EUV, localized at *x* < 0. In contrast, the high electron density mainly existed at *x* > 0. The high *T*_e_ and the high *n*_e_ were simultaneously achieved in only the small area at approximately *x* = 0 [Fig. 4(d) and (g)].

As is apparent from Fig. 4(d) and (e), a large difference in the electron density profile existed between the 1.3 μs and 2.0 μs plasmas. It should be noted that the *y* direction corresponded to the radial one. The plasma also expanded in the radial direction after the pre-pulse laser irradiation. Hence, the Sn density near the *x* axis decreased, and a hollow density profile was formed in the case of the 2.0 μs plasma. We believe that this hollow density profile of the 2.0 μs plasma was a major factor for the high conversion efficiency of 4%.

Figure 4(j) and (k) show that the maximum EUV emissivity values were almost identical. The maximum values were 1.2 and 1.4 × 10^15^ W/m^3^/eV/sr, respectively. However, the high radiation region for the 1.3 μs plasma existed in only the vicinity of the *x* axis (i.e., the axis of the laser irradiation), whereas the large emissivity region for the 2.0 μs plasma existed for the radius between 30 μm and 150 μm (i.e., 30 μm ≤ *y = r* ≤ 150 μm). The total EUV radiation was given by the volume integration of the emissivity η_EUV_. Therefore, the intense emissivity η_EUV_ at the larger radius mostly contributed to the total EUV radiation. The emitted EUV energy for the 2.0 μs plasma was much larger than that for the 1.3 μs plasma because of the higher emissivity in the region of the larger radius.

On the contrary, the region with *T*_e_ ≥ 25 eV for the 2.5 μs plasma spread in a wide area both in the *x* and radial directions. However, the maximum emissivity was only 0.9 × 10^15^ W/m^3^/eV/sr. The plasma expanded, and the electron density decreased below 4 × 10^24^ m^−3^ in the high-temperature region because of the long delay time.

We estimated the in-band EUV energies from the emissivity shown in Fig. 4(j)–(l) for the three plasma cases under the following assumptions: axial symmetry, isotropic distributions of the EUV radiation, without self-absorption, and an EUV duration of 20 ns. The obtained values were 2.1 mJ, 4.0 mJ, and 1.8 mJ for the 1.3 μs, 2.0 μs, and 2.5 μs plasmas, respectively. Although these values were calculated under the above assumptions, the calculated EUV energies approximately agree with the calorimetric measurements.

The details of the difference in the electron density and the temperature between the 1.3 μs and 2.0 μs plasmas can now be compared. A density region higher than the critical density existed for the 1.3 μs plasma at a radius of *r* ≤ 150 μm and an *x* position of 0 μm ≤ *x* ≤ 150 μm. This region has a sharp density gradient near the critical density. As a result, a high-temperature region suitable for the EUV light emission of *T*_e_ ≥ 25 eV and a high-electron density region (4 × 10^24^ m^−3^ ≤ *n*_e_ ≤ 8 × 10^24^ m^−3^) existed separately. The EUV radiation region was indeed small not only in the radial direction but also in the *x* direction. On the contrary, both a wide region of high electron temperature *T*_e_ ≥ 25 eV and high electron density (4 × 10^24^ m^−3^ ≤ *n*_e_ ≤ 8 × 10^24^ m^−3^) existed for the 2.0 μs plasma not only in the *x*-direction but also in the radial direction. Most importantly, the regions of high electron temperature and high density largely overlapped in both directions. In other words, the large volume of the good parameter range was the reason for the high conversion efficiency. Note that η_EUV_ has a strong nonlinearity with *T*_e_, as shown in Fig. 15(d) in ref.^12^.

Here we briefly describe the expansion of the droplets. Figures 1(c) and 4(a),(c) show that the spatial distributions and the sizes of the tin fragments were inhomogeneous. However, we have confirmed good reproducibility of their macroscopic behaviors, e.g., the time evolution of the diameters and the whole images of the expanding target.

Accordingly, collective Thomson scattering measurements were applied to laser-produced EUV light source plasmas. For the first time, the detailed measurements of the ion component spectra resulted in the time-resolved two-dimensional spatial profiles of *n*_e_, *T*_e_, and *Z*. The following results were experimentally obtained:

1. the two-dimensional plasma profiles of the electron density and temperature significantly changed with the delay time between the pre-pulse and main lasers; and
2. an electron density of (4–8) × 10^24^ m^−3^ and an electron temperature of ≥25 eV were simultaneously observed for the highest CE in the largest plasma volume (i.e., a good plasma structure was obtained).

The formation of this good electron density [(4–8) × 10^24^ m^−3^] and temperature (≥25 eV) profile may outline further improvements toward a higher CE in the future. We believe that these straightforward results obtained from the measured plasma parameters are very useful to further understand and optimize laser-produced plasma light sources for EUV emission.

## Method

### Collective Thomson scattering

Here, the principle of the CTS is briefly described^22,23^. The predicted Thomson scattering spectra from the EUV light source plasmas are in the collective regime when a visible probe laser is used (i.e., the scattering parameter α is larger than 1 [α = (*k*λ_D_)^−1^], where λ_D_ is the Debye length, and *k* is the absolute value of the differential scattering vector defined as ***k*** = ***k***_s_ − ***k***_i_; ***k***_i_ and ***k***_s_ are the wavevectors of the incident probe laser and the scattered light, respectively). The Thomson scattering spectrum in this regime comprises both an electron and an ion component^24,25^. Considering the strong background radiation from the plasma, we focused on only the ion component, for which we expected large signal-to-noise ratios against the background radiation even for a small probe-laser energy to avoid plasma heating^26,27^. The ion component spectrum reflects the ion acoustic wave frequency ω_ac_ = *k* [α^2^/(1 + α) (*ZκT*_e_ + 3*κT*_i_)/*m*_i_)]^1/2^, where *κ* is the Boltzmann constant, *m*_i_ is the ion mass, and *T*_i_ is the ion temperature. The spectrum exhibits two peaks (i.e., ion features with a dip between them). The wavelength separation 2Δλ_peak_ of the two peaks is related to the probe laser wavelength λ_0_ and ω_ac_ by Δλ_peak_ = λ_0_^2^ω_ac_/(2πc), where *c* is the speed of light. *ZT*_e_ and *T*_i_ are obtained from the width Δλ_peak_ and the spectral shape, which is characterized by ion acoustic wave damping^15,22^. In addition, *n*_e_ is determined by an absolute calibration of the CTS system because the scattered light intensity is proportional to the electron density. All of the plasma parameters (i.e., *T*_e_, *n*_e_, and *Z*) are then determined assuming *T*_e_ = *T*_i_.

The CTS was applied to various laser-produced plasmas (LPPs)^28–31^. However, a special challenge for the EUV light source plasmas is the very small wavelength separation of the ion features of ~100 pm at λ_0_ = 532 nm, which also means that the ion component is very close to the probe laser wavelength λ_0_ (i.e., 50 pm). Therefore, very high spectral resolution and stray light reduction are essential. Triple grating spectrometers are widely used for collective and noncollective Thomson scattering^26,32,33^. However, they block a wavelength range of approximately 1 nm at λ_0_ to reduce stray light (i.e., the ion component in our application is also blocked). Therefore, we built a custom spectrometer^14,17^. This spectrometer has six gratings. Four gratings are used for stray light reduction, while the other two gratings are utilized for wavelength dispersion. Thus, a spectral resolution of 12 pm and a sufficient stray-light rejection with a very narrow wavelength block range [within ±14 pm from λ_0_ ( = 532 nm)] were achieved, and the ion components from the Sn plasmas for the LPP-EUV light sources were clearly observed^14^.
